# Automated Removal of Ice Rings in Crystallography Images Using Denoising Autoencoder

**DOI:** 10.1063/4.0000969

**Published:** 2025-10-27

**Authors:** Kevin Fang, Yang Ha

**Affiliations:** 1 Stratford Preparatory, Santa Clara, CA, USA; 2 Lawrence Berkeley National Laboratory, Berkeley, CA, USA

## Abstract

X-ray crystallography is important for analyzing molecular and crystalline structures, but the presence of ice rings in diffraction images complicates accurate interpretation. Traditionally, the identification and removal of ice rings required specialized training and software, posing challenges in terms of time and expertise. This study aims to automate the removal of ice rings using a machine learning approach, specifically a denoising autoencoder. To enhance model robustness, a new training dataset was introduced, comprising crystallography images augmented with artificial rings overlaid on known data, thereby generating a target ground truth for the model. The proposed architecture consists of an Input Layer, followed by Encoders utilizing Convolutional Layers with 64 filters and a 3x3 kernel size activated by ReLU, combined with Max Pooling layers for dimensionality reduction. Decoders then utilized Upsampling layers and Convolutional Layers with ReLU activations, culminating in a Final Convolutional Layer with Sigmoid activation to reconstruct the de-ringed images. The model achieved a best loss of 0.004, indicating highly accurate reconstruction with ice rings becoming mostly invisible. This result demonstrates that the denoising autoencoder effectively removes ice rings, outperforming traditional methods in both efficiency and precision. The findings suggest that this approach can significantly streamline the crystallography analysis process, enhancing the accuracy of molecular and structural studies.

